# Supplementary material for: Splice-Junction-Based Mapping of Alternative Isoforms in the Human Proteome
Source: Cell Rep. Author manuscript; Available in PMC 2020 Jan 15. (PMC6961840; doi:10.1016/j.celrep.2019.11.026)
Supplement: 2 [file NIHMS1546469-supplement-2.zip › DF1/PXD006675/RightAtrium/RightAtrium_4_TTN_ASNEYGSVSCTATLTVTEPPK.pdf]

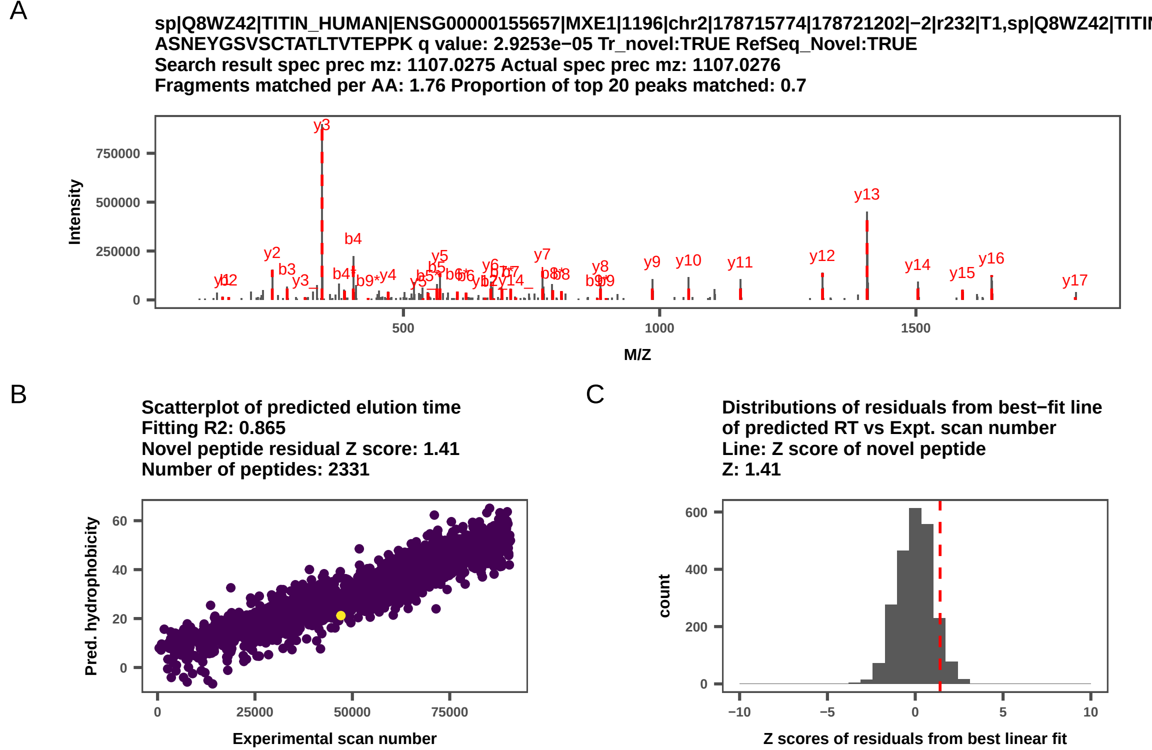
A
sp|Q8WZ42|TITIN_HUMAN|ENSG00000155657|MXE1|1196|chr2|178715774|178721202|−2|r232|T1,sp|Q8WZ42|TITI
ASNEYGSVSCTATLTVTEPPK q value: 2.9253e−05 Tr_novel:TRUE RefSeq_Novel:TRUE
Search result spec prec mz: 1107.0275 Actual spec prec mz: 1107.0276
Fragments matched per AA: 1.76 Proportion of top 20 peaks matched: 0.7
Intensity
750000
500000
250000
0
y3
y13
b4
y2
b3
y5
y7
y8
y9
y10
y11
y12
y14
y15
y16
y17
500
1000
1500
M/Z
B
Scatterplot of predicted elution time
Fitting R2: 0.865
Novel peptide residual Z score: 1.41
Number of peptides: 2331
Pred. hydrophobicity
60
40
20
0
0
25000
50000
75000
Experimental scan number
C
Distributions of residuals from best−fit line
of predicted RT vs Expt. scan number
Line: Z score of novel peptide
Z: 1.41
count
600
400
200
0
−10
−5
0
5
10
Z scores of residuals from best linear fit
